# How safe is growth hormone therapy? SAGhE and beyond

**DOI:** 10.1186/1687-9856-2013-S1-O15

**Published:** 2013-10-03

**Authors:** Jean-Claude Carel

**Affiliations:** 1 Department of Paediatric Endocrinology and Diabetology, INSERM CIE-5, Hospital and University Paris Diderot, 75019, Paris, France; 2 Centre de Référence des Maladies Endocriniennes Rares de la Croissance, AP-HP Robert Debré Hospital and University Paris Diderot, 75019, Paris, France

## 

Although short term health on growth hormone treatment has been extensively studied, little is known of the long-term health of subjects treated with growth hormone in childhood. Approximately 40 000 children are treated with growth hormone in the European Union and several hundred thousands in the world and it is essential to address this important question. SAGhE is an European consortium aimed at addressing this question as well as to gain further insight in the long term effect of growth hormone both in terms of height and of significance on quality of life. Eight countries participate in SAGhE: Belgium, France, Germany, Italy, Netherlands, Sweden, Switzerland, United Kingdom and have recruited approximately 25 000 patients who were treated for the most part in the late 80's and 90's and who are currently adults. The results of the SAGhE study as a whole are expected to be available to the investigators at the end of 2012 and will be disseminated in 2013.

However, the study did not start synchronously in all countries and results from France and from 3 other countries have been made disseminated in late 2010 and published in early 2012. In the report from France, 6928 children with idiopathic isolated GH deficiency (n = 5162), neurosecretory dysfunction (n = 534) idiopathic short stature (n = 871) or born short for gestational age (n = 335) who started treatment between 1985 and 1996 were followed on vital status up to September 2009 for 94.7% of the patients. In this population of patients, all-cause mortality was increased with a standardized mortality ratio (SMR) of 1.33. 95% CI 1.08–1.64). In multivariate analysis adjusted for height, the use of GH doses >50 µg/kg/day was associated with mortality rates using external and internal references (SMR 2.94, 95%CI 1.22–7.07, HR 2.79, 95%CI 1.14–6.82). All type cancer-related mortality was not increased. Bone tumor-related mortality was increased (SMR 5.00, 95%CI 1.01–14.63). An increase in mortality due to diseases of the circulatory system (SMR 3.07, 95%CI 1.40–5.83), subarachnoid or intracerebral hemorrhage (SMR 6.66, 95%CI 1.79–17.05) was observed. Mortality rates were therefore increased in this population of adults treated as children with recombinant GH, particularly in those who had received the highest doses.

In a parallel report, 3 additional countries (Belgium, Netherlands, Sweden) the cause of death from a cohort of 2543 patients could be identified and no cancer- or cardiovascular disease-related deaths were found. These results highlight the need for additional studies of long-term mortality and morbidity after GH treatment in childhood but do not change the risk benefit balance of growth hormone treatments, after careful evaluation by Medicine Agencies. Consolidated results of the SAGhE study are expected to be available at the end of 2012 and to be disseminated during 2013.

